# A fresh look at extended-duration anticoagulation after venous thromboembolism

**DOI:** 10.1590/1677-5449.202601062

**Published:** 2026-10-09

**Authors:** Suzanna Maria Viana Sanches, Monique Magnavita Borba da Fonseca Cerqueira, Marcos Arêas Marques

**Affiliations:** 1 Hospital Geral Ernesto Simões Filho – HGESF, Salvador, BA, Brasil.; 2 Universidade do Estado da Bahia – UNEB, Salvador, BA, Brasil.; 3 Universidade Federal do Estado do Rio de Janeiro – UNIRIO, Hospital Universitário dos Servidores do Estado – HUSE, Rio de Janeiro, RJ, Brasil.; 4 Universidade do Estado do Rio de Janeiro – UERJ, Hospital Universitário Pedro Ernesto – HUPE, Rio de Janeiro, RJ, Brasil.

Prins et al.^1^ conduced an integrated analysis of the data from two large randomized clinical trials (RCTs) — the EINSTEIN-EXTENSION^2^ and the EINSTEIN-CHOICE studies^3^ — with the primary objective of estimating the risk of recurrence of venous thromboembolism (VTE) in patients who had completed from 6 to 12 months of initial treatment, categorizing them according to the profiles of the risk factors associated with the index event (unprovoked VTE or VTE provoked by major transient or persistent or minor transient or persistent risk factors). The authors called attention to the fact that patients with VTE associated with minor risk factors had a substantial annual risk of recurrence (7%) and, therefore, may also benefit from extended anticoagulation, in common with patients with unprovoked VTE. Moreover, the authors also observed that rivaroxaban (10 or 20 mg) reduced risk of recurrence by more than 75% compared with placebo, irrespective of the patient’s risk profile, and without increasing rates of major bleeding compared to aspirin or placebo.

After publication of this study, extending anticoagulant treatment began to be considered for VTE patients beyond those with no clear trigger risk factor.

The HI-PRO study^4^ (Extended-Duration Low-Intensity Apixaban to Prevent Recurrence in High-Risk Patients with Provoked Venous Thromboembolism) was an RCT designed to address the uncertainty surrounding the ideal duration of anticoagulation in patients with provoked VTE who have persistent risk factors. Traditionally, guidelines recommend just 3 months of treatment for provoked VTE, but associated with transitory risk factors. This double-blind and placebo controlled RCT assessed 600 adults who had had a VTE provoked by transitory risk factors (surgery, trauma, immobility, or acute disease), who had completed at least 3 months’ anticoagulation and had at least one persistent risk factor, such as obesity [body mass index (BMI) ≥ 30 kg/m^2^]. The 300 patients who were given 2.5 mg apixaban twice a day for 12 months exhibited an 87% reduction in risk of recurrence (hazard ratio [HR]: 0.13; 95%CI 0.04–0.36; P < 0.001), with no increase in the risk of major bleeding, compared with the placebo group. These results demonstrate that apixaban at a prophylactic dose, administered for 12 months, was effective for prevention of recurrence in patients with provoked VTE associated with minor persistent risk factors. Moreover, they show that the traditional classification of VTE as "provoked" or "unprovoked" is insufficient to determine anticoagulation duration, since presence of persistent risk factors, even those considered minor, identifies patients with a high risk of recurrence who could benefit from extended duration anticoagulant therapy. Rather than a dichotomous classification, the emerging concept is of a continuous spectrum of risk of recurrence, determined by persistence and intensity of the predisposing factors, rather than by a merely binary classification.

The RENOVE study^5^ (Extended treatment of venous thromboembolism with reduced-dose versus full-dose direct oral anticoagulants in patients at high risk of recurrence) was a multicenter RCT conducted at 47 hospitals in France that compared the efficacy and safety of prophylactic doses versus therapeutic doses of apixaban or rivaroxaban for extended duration treatment of 2,768 adult patients with acute symptomatic VTE who had already completed from 6 to 24 months of initial treatment with therapeutic dosages and had indications for extended anticoagulation. The composite outcome (recurrence of VTE + bleeding) favored prophylactic doses (HR: 0.67) and the all-causes mortality rate was smaller in the prophylactic dose group (4.3% vs. 6.1%). While the prophylactic dose did not achieve the formal statistical criterion for non-inferiority of efficacy, it did achieve a 39% reduction in clinically relevant bleeding, resulting in a superior net benefit for the majority of patients. However, the authors suggest that although the prophylactic dose was an option for extended therapy in view of the substantial reduction in bleeding, the therapeutic dose may still be preferable in specific subgroups, such as in patients with persistent risk factors, such as obesity, or in those who have had a severe VTE episode, suggesting that care should be exercised before universally adopting the reduced dose.

These studies have brought significant advances for individualization of extended anticoagulation in VTE patients, challenging traditional practices and refining patient selection. In conjunction, they highlight three essential fundamentals: the dichotomous classification of VTE as "provoked" or "unprovoked" is insufficient to determine the duration of anticoagulation; extended anticoagulation with direct oral anticoagulants (DOACs) is associated with very low rates of major bleeding, making it a viable option for patients at elevated risk of recurrence; and the dose used for extended therapy (prophylactic or therapeutic) can be individualized according to each patient’s risk of recurrence and risk of bleeding.

Nevertheless, in clinical practice one relevant question remains unanswered: which patients have sufficient net benefit to justify years of treatment? The decision remains dependent on integration of the risks of recurrence and bleeding, the patient’s preferences and values, estimated survival, and the impact on quality of life, which are aspects that are not yet covered by current stratification models.

The HI-PRO^4^ and RENOVE^5^ studies converge to the conclusion that it is necessary to personalize duration of treatment on the basis of persistence of risk factors, but they diverge on the universality of dose reduction, with the RENOVE^5^ study suggesting a more cautious approach to patients with high risk of recurrence.
