# Supplementary material for: A fresh look at extended-duration anticoagulation after venous thromboembolism
Source: J Vasc Bras. 2026 Oct 9;25:e20260106. doi: 10.1590/1677-5449.202601062 (PMC13671011; doi:10.1590/1677-5449.202601062)
Supplement: Translation

# Translation: Um novo olhar sobre a extensão da anticoagulação no tromboembolismo venoso

**DOI:** 10.1590/1677-5449.202601061

**Authors:** Suzanna Maria Viana Sanches, Monique Magnavita Borba da Fonseca Cerqueira, Marcos Arêas Marques

**Affiliations:** 1 Hospital Geral Ernesto Simões Filho – HGESF, Salvador, BA, Brasil.; 2 Universidade do Estado da Bahia – UNEB, Salvador, BA, Brasil.; 3 Universidade Federal do Estado do Rio de Janeiro – UNIRIO, Hospital Universitário dos Servidores do Estado – HUSE, Rio de Janeiro, RJ, Brasil.; 4 Universidade do Estado do Rio de Janeiro – UERJ, Hospital Universitário Pedro Ernesto – HUPE, Rio de Janeiro, RJ, Brasil.

O estudo de Prins et al.^1^ , uma análise integrada dos dados de dois grandes ensaios clínicos randomizados (ECRs) — a saber, o EINSTEIN-EXTENSION^2^ e o EINSTEIN-CHOICE^3^ —, teve como objetivo principal estimar o risco de recorrência do tromboembolismo venoso (TEV) em pacientes que completaram de 6 a 12 meses de tratamento inicial, categorizando-os de acordo com o perfil dos fatores de risco associados ao evento índice (TEV não provocado ou provocado por fatores de risco maiores ou menores, transitórios ou persistentes). Os autores chamaram atenção para o fato de que pacientes com TEV associado a fatores de risco menores apresentaram um risco anual substancial de recorrência (7%) e, portanto, poderiam se beneficiar da anticoagulação estendida de forma semelhante aos pacientes com TEV não provocado. Além disso, os autores observaram que a rivaroxabana (10 ou 20 mg) reduziu o risco de recorrência em mais de 75% em comparação ao placebo, independentemente do perfil de risco do paciente, e sem aumento das taxas de sangramento maior quando comparada ao ácido acetilsalicílico ou ao placebo.

A partir desse estudo, passou-se a considerar a extensão do tratamento anticoagulante para além dos pacientes com TEV sem fator de risco desencadeante claro.

O estudo HI-PRO^4^ (*Extended-Duration Low-Intensity Apixaban to Prevent Recurrence in High-Risk Patients with Provoked Venous Thromboembolism*) foi um ECR desenhado para abordar a incerteza sobre a duração ideal da anticoagulação em pacientes com TEV provocado, mas que possuem fatores de riscos persistentes. Tradicionalmente, as diretrizes recomendam apenas 3 meses de tratamento para o TEV provocado, porém associado a fatores de riscos transitórios. Esse ECR, que foi duplo-cego e controlado por placebo, avaliou 600 adultos que tiveram um TEV provocado por fatores de risco transitórios (cirurgia, trauma, imobilidade ou doença aguda), que já haviam completado pelo menos 3 meses de anticoagulação e possuíam ao menos um fator de risco persistente associado, como obesidade [índice de massa corpórea (IMC) ≥ 30 kg/m^2^]. Os 300 pacientes que receberam apixabana 2,5 mg duas vezes ao dia por 12 meses apresentaram redução de 87% no risco de recorrência [*hazard ratio* (HR): 0,13; IC95% 0,04–0,36; P < 0,001), sem aumento do risco de sangramento maior, em comparação ao grupo placebo. Esses resultados demonstram que a apixabana em dose profilática, administrada por 12 meses, foi eficaz na prevenção de recorrências em pacientes com TEV provocado associado a fatores de risco menores persistentes. Além disso, eles reforçam que a classificação tradicional do TEV como "provocado" ou "não provocado" é insuficiente para determinar a duração da anticoagulação, uma vez que a presença de fatores de risco persistentes, ainda que considerados menores, identifica pacientes com elevado risco de recorrência que podem se beneficiar da terapia anticoagulante estendida. Em vez de uma classificação dicotômica, emerge o conceito de um espectro contínuo de risco de recorrência, determinado pela persistência e intensidade dos fatores predisponentes e não por uma classificação binária apenas.

O estudo RENOVE^5^ (*Extended treatment of venous thromboembolism with reduced-dose versus full-dose direct oral anticoagulants in patients at high risk of recurrence*) foi um ECR e multicêntrico realizado em 47 hospitais na França, que comparou a eficácia e a segurança de doses profiláticas *versus* doses terapêuticas de apixabana ou rivaroxabana no tratamento estendido de 2.768 pacientes adultos com TEV sintomático agudo que já haviam completado entre 6 e 24 meses de tratamento inicial com dose terapêutica e possuíam indicação para anticoagulação estendida. O desfecho composto (recorrência de TEV + sangramentos) favoreceu as doses profiláticas (HR: 0,67), e a taxa de mortalidade por todas as causas foi menor no grupo de dose profilática (4,3% *vs.* 6,1%). Embora a dose profilática não tenha atingido formalmente o critério estatístico de não inferioridade em eficácia, ela proporcionou uma redução de 39% nos sangramentos clinicamente relevantes, resultando em um benefício clínico líquido superior para a maioria dos pacientes. Porém, os autores sugerem que, apesar da dose profilática ter sido uma opção na extensão devido à redução substancial de sangramentos, a dose terapêutica pode ainda ser preferível em subgrupos específicos, como em pacientes com fatores de risco persistentes, como obesidade, ou naqueles que tiveram um episódio de TEV grave, sugerindo cautela na extensão com a dose reduzida de forma universal.

Esses estudos trouxeram avanços significativos para a individualização da anticoagulação estendida no TEV, desafiando práticas tradicionais e refinando a seleção dos pacientes. Em conjunto, eles destacaram três pontos fundamentais: a insuficiência da classificação dicotômica do TEV em "provocado" e "não provocado" para determinar a duração da anticoagulação; a demonstração de que a anticoagulação estendida com anticoagulantes orais diretos (DOACs) está associada a taxas muito baixas de sangramento maior, tornando-se uma opção viável para pacientes com elevado risco de recorrência; e que a dose utilizada durante a fase de extensão (profilática ou terapêutica) pode ser individualizada de acordo com o risco de recorrência e de sangramento de cada paciente.

Contudo, permanece aberta uma questão relevante na prática clínica: quais são os pacientes que apresentam benefício líquido suficiente para justificar anos de tratamento. A decisão continua dependente da integração entre riscos de recorrência e sangramento, preferências e valores do paciente, sobrevida estimada e impacto na qualidade de vida, aspectos esses que ainda não são contemplados pelos modelos atuais de estratificação.

Os estudos HI-PRO^4^ e RENOVE^5^ convergem para a necessidade de personalizar a duração do tratamento com base na persistência de fatores de risco, porém divergem sobre a universalidade da redução da dose, com o estudo RENOVE^5^ sugerindo uma abordagem mais cautelosa em perfis de altíssimo risco de recorrência.
